# Tetrabenazine Versus Deutetrabenazine for Huntington's Disease: Twins or Distant Cousins?

**DOI:** 10.1002/mdc3.12483

**Published:** 2017-03-29

**Authors:** Filipe B. Rodrigues, Gonçalo S. Duarte, João Costa, Joaquim J. Ferreira, Edward J. Wild

**Affiliations:** ^1^ Huntington's Disease Center Institute of Neurology University College London London United Kingdom; ^2^ Laboratory of Clinical Pharmacology and Therapeutics Faculty of Medicine University of Lisbon Lisbon Portugal; ^3^ Clinical Pharmacology Unit Instituto de Medicina Molecular Lisbon Portugal; ^4^ Cochrane Movement Disorders Group Faculty of Medicine University of Lisbon Lisbon Portugal; ^5^ Center for Evidence‐Based Medicine Faculty of Medicine University of Lisbon Lisbon Portugal

**Keywords:** deutetrabenazine, Huntington's disease, indirect comparison, meta‐analysis, tetrabenazine

## Abstract

**Background:**

Tetrabenazine is the only US Food and Drug Administration‐approved drug for Huntington's disease, and deutetrabenazine was recently tested against placebo. A switching‐trial from tetrabenazine to deutetrabenazine is underway, but no head‐to‐head, blinded, randomized controlled trial is planned. Using meta‐analytical methodology, the authors compared these molecules.

**Methods:**

RCTs comparing tetrabenazine or deutetrabenazine with placebo in Huntington's disease were searched. The authors assessed the Cochrane risk‐of‐bias tool, calculated indirect treatment comparisons, and applied the Grading of Recommendations Assessment, Development, and Evaluation (GRADE) framework.

**Results:**

The evidence network for this report comprised 1 tetrabenazine trial and 1 deutetrabenazine trial, both against placebo. Risk of bias was moderate in both. Participants in the tetrabenazine and deutetrabenazine trials did not differ significantly on motor scores or adverse events. Depression and somnolence scales significantly favored deutetrabenazine.

**Conclusion:**

There is low‐quality evidence that tetrabenazine and deutetrabenazine do not differ in efficacy or safety. It is important to note that these results are likely to remain the only head‐to‐head comparison between these 2 compounds in Huntington's disease.

Huntington's disease (HD) is a hereditary neurodegenerative condition characterized by progressive motor, cognitive, and behavioral dysfunction.^1^ Tetrabenazine (TBZ) is the only US Food and Drug Administration‐approved drug for chorea in HD, and is usually taken 3 times daily. Although it was developed to treat psychosis, it was later found to ease hyperkinetic movement disorders, including chorea, tics, tardive dyskinesia, and dystonia, although, in the United States, it is licensed only for treating chorea.^2^ Unlike classical neuroleptics, this compound depletes presynaptic dopamine by blocking vesicular monoamine transporter type 2 (VMAT2).^3^ Deutetrabenazine (DEU), a structurally related molecule with deuterium (a heavy hydrogen isotype) placed at key positions, was recently tested successfully against placebo.^4^ Deuteration prolongs half‐life, reduces metabolism variability, and is proposed to translate into less frequent dosing, a lower daily dose, and improved tolerability. The FIRST‐HD study (http://clinicaltrials.gov identifier NCT01795859) has been interpreted as offering support for similar efficacy of DEU with respect to TBZ, but with fewer adverse effects and easier dosing.^5^ An unmasked switching design trial from TBZ to DEU (ARC‐HD; http://clinicaltrials.gov identifier NCT01897896) is underway, but no head‐to‐head, blinded, randomized trial is planned. Therefore, we set out to compare TBZ and DEU indirectly using meta‐analysis methodology.

## Materials and Methods

Our study protocol was registered (PROSPERO CRD42016049199) following the PRISMA‐NMA framework.^6^ We included randomized controlled trials that compared TBZ or DEU with placebo in patients with HD. The following outcome domains were studied: motor, depression, somnolence, and adverse events (AEs). Severe AEs (SAEs) were classified according to the primary studies, although neither reported a formal definition of an SAE. References were searched in the MEDLINE, Embase, an SAE and CENTRAL databases, the combining (Huntington) with (tetrabenazine OR deutetrabenazine) and applying the Cochrane Highly Sensitive Search Strategy for identifying randomized trials. Studies were evaluated using the Cochrane risk‐of‐bias tool. Study selection, data collection, and appraisal were done independently in duplicate. Continuous and dichotomous variables were presented as mean differences (MDs) and odds ratios, respectively, both with 95% confidence intervals (95% CIs). Indirect treatment comparison meta‐analyses between TBZ and DEU were calculated based on a common comparator using the Bucher method.^7^

Confidence in cumulative evidence was assessed using the Grading of Recommendations Assessment, Development, and Evaluation (GRADE) Working Group guidelines.^8^ These comprise a widely endorsed tool to assess the quality of studies contributing to meta‐research that takes into account the domains: risk of bias, inconsistency, indirectness, imprecision, and publication bias, and classifies evidence from high to very low quality, as follows:

- High quality: We are very confident that the true effect lies close to that of the estimate of the effect;
- Moderate quality: We are moderately confident in the effect estimate; the true effect is likely to be close to the estimate of the effect, but there is a possibility that it is substantially different;
- Low quality: Our confidence in the effect estimate is limited; the true effect may be substantially different from the estimate of the effect; and
- Very low quality: We have very little confidence in the effect estimate; the true effect is likely to be substantially different from the estimate of effect.

A sample‐size calculation for a 1:1, parallel equivalence trial was calculated using Stata 14.0 software (Austin, Texas) assuming 80% power, 10% dropout, 0.05 alpha, a standard deviation of 3.5 points on the Unified Huntington's Disease Rating Scale (UHDRS) chorea subscale score, and 20% margin of equivalence of TBZ effect (5 UHDRS chorea score points).^9^

## Results

**Figure 1:**
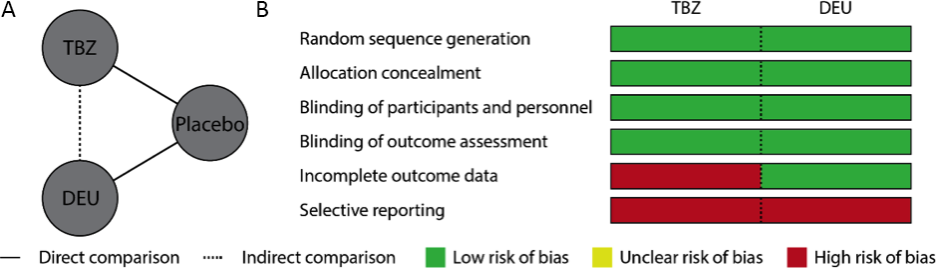
A: Indirect comparison model and (B) the risk of bias in source studies. With 1 or 2 of 6 domains at high risk of bias, the overall risk of bias for each of these studies was classified as moderate. TBZ, tetrabenazine; DEU, deutetrabenazine.

In total, 131 references were retrieved, and 2 studies were included.^4^, ^9^ Our evidence network, describing how the included studies related to one another, comprised 1 trial that tested TBZ (TETRA‐HD; n = 84) and another that tested DEU (FIRST‐HD; n = 90), both against placebo (Fig. 1A). The overall risk of bias was moderate in both studies because of attrition and reporting bias (Fig. 1B). In the TBZ arm of TETRA‐HD, proportionally more participants withdrew from the study than in the placebo arm; and, in both studies, several important outcome measures, such as quality of life, were missing. In other respects (random sequence generation, allocation concealment, blinding of patient and participants, blinding of outcome assessments, and incomplete outcome data for the DEU trial), the studies were at low risk of bias.

After a detailed review of the methodologies and trial populations, we considered that the included studies were methodologically and clinically similar and comparable on effect modifiers, confirming the transitivity assumption needed to calculate an unbiased, indirect estimate of TBZ versus DEU.

Both TBZ and DEU had a mild effect on chorea versus placebo (5.0 and 4.4 point improvement in the UHDRS chorea score, respectively) and did not differ significantly on UHDRS the chorea score (MD −1.00; 95% CI −3.04, 1.04) or the total motor score (MD 0.70; 95% CI −3.72, 5.12) (Table 1). Depression and somnolence, which were evaluated using rating scales, favored DEU significantly over TBZ in both clinical domains (MD 0.94; 95% CI 0.88–1.00; and MD 2.10; 95% CI 0.08–4.12, respectively) (Table 1). The odds of specific AEs did not differ significantly between interventions (Table 2). The required sample size calculated for a 1:1, parallel, head‐to‐head equivalence trial of TBZ versus DEU was 608 participants.

**Table 1:** Outcomes for direct and indirect comparisons

Outcome	Mean difference (95% CI)		
	Direct comparisons^a^		Indirect comparisons^b^
	TBZ‐placebo	DEU‐placebo	TBZ‐DEU
UHDRS chorea score	−3.5 (−5.2, −1.9)^c^	−2.5 (−3.7, −1.3)^d^	−1.00 (−3.04, 1.04)
UHDRS total motor score	−3.3 (−7.0, 0.3)	−4.0 (−6.5, −1.5)^d^	0.70 (−3.72, 5.12)
Depression scale	0.76 (0.71, 0.81)^e^	−0.18 (−0.22, −0.14)^d^	0.94 (0.88, 1.00)^d^
Epworth Sleepiness Scale	1.8 (0.3, 3.4)^e^	−0.3 (−1.6, 1.0)^d^	2.10 (0.08, 4.12)^d^

CI, confidence interval; TBZ, tetrabenazine; DEU, deutetrabenazine; UHDRS, Unified Huntington's Disease Rating Scale.

a For direct comparisons, the values presented are the difference between active treatment and placebo in the mean change in score reported in each individual study. In each case, positive values are in favor of placebo, and negative values are in favor of active treatment.

b For indirect comparisons, the values represent the difference between TBZ and DEU in the mean change in score. Here, positive values are in favor of DEU, and negative values are in favor of TBZ. In the TBZ study, UHDRS chorea scores were adjusted to baseline values and site, and depression was summarized using the Hedges g effect size from the Hamilton Depression Scale. In the DEU study, UHDRS chorea scores were adjusted to baseline values only, and depression was summarized using the Hedges g effect size from the Hospital and Anxiety Depression Scale depression subscale.

c Significantly favors TBZ.

d Significantly favors DEU.

e Significantly favors placebo.

**Table 2:** Adverse events for direct and indirect comparisons

Adverse event	Odds ratio (95% CI)^a^		
	Direct comparisons^b^		Indirect comparisons^c^
	TBZ‐placebo	DEU‐placebo	TBZ‐DEU
Serious adverse events, as defined by study authors	5.44 (0.28, 104.49)	1.00 (0.06, 16.50)	5.44 (0.09, 322.08)
Somnolence	13.32 (1.67, 106.07)^d^	2.69 (0.49, 14.64)	4.95 (0.34, 72.37)
Diarrhea	0.72 (0.15, 3.46)	9.87 (0.52, 188.88)	0.07 (0.03, 2.06)
Insomnia	21.84 (1.25, 380.62)^d^	1.54 (0.24, 9.66)	14.18 (0.47, 426.77)
Fatigue	1.86 (0.54, 6.37)	1.54 (0.24, 9.66)	1.21 (0.31, 11.14)
Falls	1.30 (0.36, 4.64)	0.48 (0.08, 2.74)	2.71 (0.31, 23.98)
Depression	11.15 (0.62, 200.33)	0.65 (0.10, 4.10)	17.15 (0.55, 531.90)

CI, confidence interval; TBZ, tetrabenazine; DEU, deutetrabenazine.

a All values are odds ratios with 95% CIs in parentheses, with 1 indicating absence of difference. CIs not spanning 1 indicate a statistically significantly altered odds.

b For direct comparisons, values greater than 1 indicate an increased odds in the active treatment arm.

c For indirect comparisons, values greater than 1 indicate an increased odds for TBZ.

d Significantly favors placebo.

## Discussion

Our indirect comparison, as assessed according to the GRADE framework, shows that there is low‐quality evidence that TBZ and DEU do not differ in efficacy and safety. DEU appears significantly less prone to depressive symptoms and somnolence, but this observation, which was drawn from indirect analysis of a restricted evidence network, requires validation in a direct, suitably designed trial.

Our analysis must be interpreted with caution overall, because indirect comparisons only provide observational evidence: the power of hypothesis testing relies on between‐study heterogeneity, which thankfully was minimal in this case. Furthermore, the power of our computation is limited by the evidence network sample size.^10^

If DEU receives licensing authorization, then long‐term, phase 4 studies and real‐world practice will provide further information on the clinical utility of DEU. Nonetheless, our analysis raises concerns for informed clinical decision making in HD: no clinical trial has recruited over 600 participants; and, to our knowledge, only 1 ongoing trial seeks to compare TBZ and DEU directly: ARC‐HD, whose nonrandomized, open‐label, switching design carries a risk of selection, detection, and performance bias. Therefore, the present study seems likely to remain the only feasible and realistic, blinded, head‐to‐head comparison between TBZ and DEU in HD.

## Author Roles

1. Research Project: A. Conception, B. Organization, C. Execution; 2. Statistical Analysis: A. Design, B. Execution, C. Review and Critique; 3. Manuscript Preparation: A. Writing the First Draft, B. Review and Critique.

F.B.R.: 1A, 1B, 1C, 2A, 2B, 3A

J.J.F.: 1A, 3B

E.J.W.: 1A, 3B

G.S.D.: 1B, 1C, 2B

J.C.: 2C, 3B

## Disclosures

**Ethical Compliance Statement:** We confirm that we have read the Journal's position on issues involved in ethical publication and affirm that this work is consistent with those guidelines.

**Funding Sources and Conflict of Interest:** Filipe B. Rodrigues, Joaquim J. Ferreira, and Edward J. Wild are investigators on a TEVA‐sponsored trial of another drug, laquinimod. None have received any personal payments or salary contributions for this work, nor have they been involved in any TEVA‐sponsored trial of pridopidine. Filipe B. Rodrigues, Joaquim J. Ferreira, and Edward J. Wild are supported by CHDI Foundation. Joaquim J. Ferreira received research funds from GlaxoSmithKline, Grunenthal, Fundação MSD (Portugal), TEVA, MSD, Allergan, Ipsen, Novartis, Medtronic. Edward J. Wild is supported by the Medical Research Council and received research funds from GlaxoSmithKline Foundation. Filipe B. Rodrigues and Gonçalo S. Duarte are external editors of the Cochrane Movement Disorders Group, and João Costa is the editor of Cochrane Movement Disorders Group.

**Financial disclosures for the previous 12 months:** Joaquim J. Ferreira has speaker and consultant relationships with GlaxoSmithKline, Novartis, TEVA, Lundbeck, Solvay, Abbott, BIAL, Merck‐Serono, Merz, Ipsen, Biogen, and Sunovion Pharmaceuticals, none of which has a known, specific interest in the submitted work. Edward J. Wild has participated in scientific advisory boards with Hoffmann‐La Roche Ltd., Ionis, Shire, GlaxoSmithKline, and Wave Life Sciences. All honoraria were paid through UCL Consultants Ltd., a wholly owned subsidiary of UCL. Filipe B. Rodrigues, Gonçalo S. Duarte, and João Costa reported no sources of funding and no conflicts of interest.
